# The impact of coenzyme Q_10_ on metabolic and cardiovascular disease profiles in diabetic patients: A systematic review and meta‐analysis of randomized controlled trials

**DOI:** 10.1002/edm2.118

**Published:** 2020-03-14

**Authors:** Phiwayinkosi V. Dludla, Tawanda M. Nyambuya, Patrick Orlando, Sonia Silvestri, Vuyolwethu Mxinwa, Kabelo Mokgalaboni, Bongani B. Nkambule, Johan Louw, Christo J. F. Muller, Luca Tiano

**Affiliations:** ^1^ Biomedical Research and Innovation Platform South African Medical Research Council Tygerberg South Africa; ^2^ Department of Life and Environmental Sciences Polytechnic University of Marche Ancona Italy; ^3^ School of Laboratory Medicine and Medical Sciences College of Health Sciences University of KwaZulu‐Natal Durban South Africa; ^4^ Department of Health Sciences Faculty of Health and Applied Sciences Namibia University of Science and Technology Windhoek Namibia; ^5^ Department of Biochemistry and Microbiology University of Zululand KwaDlangezwa South Africa; ^6^ Division of Medical Physiology Faculty of Health Sciences Stellenbosch University Tygerberg South Africa

**Keywords:** cardiovascular diseases, coenzyme Q_10_, diabetes mellitus, heart failure, metabolic syndrome, oxidative stress

## Abstract

**Aims:**

Coenzyme Q_10_ (CoQ10) is well known for its beneficial effects in cardiovascular disease (CVD); however, reported evidence has not been precisely synthesized to better inform on its impact in protecting against cardiovascular‐related complications in diabetic patients.

**Materials and Methodology:**

The current meta‐analysis included randomized controlled trials published in the past 5 years reporting on the effect of CoQ10 on metabolic and CVD‐related risk profiles in individuals with diabetes or metabolic syndrome. We searched electronic databases such as MEDLINE, Cochrane Library, Scopus and EMBASE for eligible studies. In addition to assessing the risk of bias and quality of evidence, the random and fixed‐effect models were used to calculate the standardized mean difference and 95% confidence intervals for metabolic parameters and CVD outcomes.

**Results:**

Overall, 12 studies met the inclusion criteria, enrolling a total of 650 patients. Although CoQ10 supplementation did not statistically affect all metabolic profiles measured, it significantly reduced CVD‐risk‐related indexes such as total cholesterol and low‐density lipoprotein (LDL) levels in diabetic patients when compared to those on placebo [SMD = 0.13, 95% CI (0.03; 0.23), Chi^2^ = 43.62 and *I* ^2^ = 29%, *P* = .07].

**Conclusions:**

The overall results demonstrated that supplementation with CoQ10 shows an enhanced potential to lower CVD risk in diabetic patients by reducing total cholesterol and LDL. Moreover, the beneficial effects of CoQ10 in lowering the CVD risk are associated with its ameliorative properties against oxidative stress and improving endothelial health.

## Graphical abstract

Although clinical evidence on the impact of coenzyme Q10 (CoQ10) against cardiovascular diseases (CVDs) is available, such information has not been efficiently scrutinized to better inform on its protection against the diabetes‐associated CVD risk. In addition to modulating some metabolic disease‐associated parameters, the current meta‐analysis of randomized controlled trials showed that CoQ10 can protect against diabetes‐associated CVD‐related risk by lowering total cholesterol and lipoprotein to improve endothelial health in patients with diabetes and metabolic syndrome.

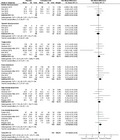

**Abbreviations**
BMI: body mass index
CoQ10: coenzyme Q_10_
CVD: cardiovascular disease
FPG: fasting plasma glucose
GRADE: Grading of Recommendations Assessment Development and Evaluation
HbA1c: glycated haemoglobin
HDL: high‐density lipoprotein
LDL: low‐density lipoprotein
NO: nitric oxide
PRISMA: Preferred Reporting Items for Systematic reviews and Meta‐Analysis
RCT: randomized controlled trials
ROS: reactive oxygen species
T2D: type 2 diabetes

## 1 INTRODUCTION

Heart failure remains the leading cause of death in diabetic patients, when compared to their nondiabetic counterparts.^1^ A combination of metabolic abnormalities is acknowledged to be responsible for enhanced susceptibility of diabetic individuals to myocardial damage. For example, in type 2 diabetes (T2D), the predominant form of diabetes that is associated with dyslipidaemia and enhanced arterial atherosclerotic buildup has been directly connected with endothelial dysfunction and subsequent increased risk of heart failure.^1^, ^2^, ^3^, ^4^ The characteristic features of diabetic dyslipidaemia include elevated plasma triglyceride and low‐density lipoprotein (LDL) concentrations, and reduced high‐density lipoprotein (HDL) levels. Such complications may arise due to increased free fatty acid flux secondary to insulin resistance and can greatly impair myocardial contractility, which eventually leads to reduced cardiac efficiency.^5^, ^6^ Currently, of major concern has been the limited capacity of available therapies to protect diabetic patients against the rising cardiovascular disease (CVD)‐related comorbidities.

Although currently used therapies like statins can control dyslipidaemic complications, their long‐term use has been associated with reduced endogenous levels of ubiquinone or coenzyme Q_10_ (CoQ10).^7^, ^8^ In fact, it has been established that a diabetic heart already displays significantly reduced endogenous CoQ10 levels, when compared to the nondiabetic counterpart.^9^ This may further explain the continued rise in CVD‐related deaths in diabetic patients since CoQ10 is known to act as an important antioxidant to protect against dyslipidaemia‐induced oxidative stress and inflammation,^10^, ^11^ some of the major consequences implicated in enhanced diabetes‐induced myocardial damage. Beyond its antioxidant properties, CoQ10 remains a crucial component of the mitochondrial electron transport chain that plays an essential role in facilitating the production of adenosine triphosphate through its involvement in redox reactions.^12^ Consistently, administration of CoQ10 has been correlated with improved endothelial function in patients with and without established CVDs.^10^

Despite an increase in the number of studies reporting on the beneficial effects of CoQ10 supplementation in improving endothelial function in humans,^10^ available evidence remains inconclusive regarding its impact on improving CVD outcomes in those with diabetes. Thus, this systematic review and meta‐analysis updates our current understanding on the cardio‐protective effects of CoQ10, using data from randomized controlled trials (RCTs) published in the last five years.

## 2 METHODS

This systematic review and meta‐analysis was prepared in agreement with the Preferred Reporting Items for Systematic reviews and Meta‐Analysis (PRISMA) guidelines.^13^ Accordingly, File S1 provides a PRISMA checklist for this systematic review and meta‐analysis. Furthermore, to deliver transparency in the review process and avoid publication bias, this meta‐analysis, the International prospective register of systematic reviews (PROSPERO), was thoroughly searched and found no similar review registered on the current topic.

### 2.1 Strategy to search RCTs

For study inclusion, a comprehensive search was conducted using electronic databases such as MEDLINE, Cochrane Library, Scopus and EMBASE from inception up to 30 September 2019. This was done independently by two reviewers, PVD and TMN, whilst BBN as a third reviewer was consulted for arbitration. The primary search was limited to RCTs, published in the past 5 years to capture recent development in the topic, reporting the use of CoQ10 supplementation in individuals with diabetes or metabolic syndrome. Within the same cohort, patients receiving placebo were used as a comparative control. To optimize the search strategy, Medical Subject‐Heading (MeSH) and text words such as coenzyme Q_10_, diabetes mellitus, metabolic syndrome, hyperglycaemia, heart failure and their respective synonyms and associated words or phrases were adapted for each database used. Moreover, there were no language restrictions applied in the search strategy, whilst EndNote version 10 (Clarivate Analytics, Philadelphia, USA) was used to manage the reference list, including removing study duplicates, as previously reported.^14^

### 2.2 Inclusion and exclusion criteria

The systematic review and meta‐analysis included RCTs evaluating the impact of CoQ10 on CVD‐related outcomes in adults (>18 years) with diabetes or metabolic syndrome. Briefly, included studies were those that assessed the use of CoQ10 as an intervention, contained the comparison group on placebo, and reported on measurable CVD‐related outcomes in individuals with diabetes or metabolic syndrome. Animal studies were excluded since the main objective was to establish the impact of CoQ10 supplementation on CVD outcomes in individuals with diabetes or metabolic syndrome. Other exclusions included books, cohort or observational studies, letters and case reports, whilst reviews were only scanned for RCTs. Furthermore, studies not reporting on measurable CVD outcome or contained limited information on the methodology or results from the article were excluded.

### 2.3 Data extraction and assessment of quality

Two investigators, PVD and TMN, independently evaluated all pertinent articles and carefully selected those that were relevant. Incongruities were resolved by consulting a third investigator, BBN. The main outcome of the study was to establish the impact of CoQ10 supplementation on CVD‐related outcomes in diabetes or metabolic syndrome. Another important objective of the study was to establish whether CoQ10 supplementation affected diabetes and metabolic syndrome‐related markers such as fasting blood glucose (FPG) or insulin levels, glycated haemoglobin (Hb1Ac) and body mass index (BMI). To accomplish this, relevant data items, from each article, such as name and year of publication, the country where the study was conducted, sample and gender distribution, as well as CoQ10 dosage used and duration of intervention, were extracted by two independent investigators (VM and KM). Furthermore, VM and KM evaluated the risk of bias using the modified Downs and Black checklist, which is appropriate for both randomized and nonrandomized studies.^15^, ^16^ Any disagreements were resolved by consulting the third investigator, TMN. The overall total scores of each study was rated poor if the score was (≤13points), fair if (14‐18 points), good if (19‐23 points) and excellent if the score was (24‐27). The same investigators were also responsible for assessing the quality of evidence across the selected studies by using the Grading of Recommendations Assessment Development and Evaluation (GRADE) approach.^17^

### 2.4 Statistical analysis

The meta‐analysis and statistical analyses was performed using RevMan software (version 5.0; Cochrane Collaboration, Oxford, UK). The method established by Hozo and colleagues was used to calculate each continuous effect measure.^18^ Alternatively, to test for statistical heterogeneity, Pearson's chi‐squared test (Chi^2^) and Higgin's *I* ^2^ statistics were applied.^19^ Furthermore, to generate pooled effect estimates when substantial heterogeneity existed (*I* ^2^ > 50%), the random‐effects model was used, as previously discussed.^20^ Cohen's method was further used to interpret effect sizes, whereby a standardized mean difference of 0.2, 0.5 and 0.8 was equated to small, medium and large, respectively.^21^ Likewise, a *P*‐value < .05 was considered statistically significant, whilst interrater reliability was evaluated for both the included studies and risk of bias by means of Cohen's kappa. Here, a kappa value of <0.00 was taken as poor strength of agreement, 0.00‐0.20 as slight agreement, 0.21‐0.40 as fair agreement, 0.41‐0.60 as moderate agreement, 0.61‐0.80 as substantial agreement, and 0.81‐1.00 as perfect agreement.^22^

## 3 RESULTS

### 3.1 Study selection

**Figure 1:**
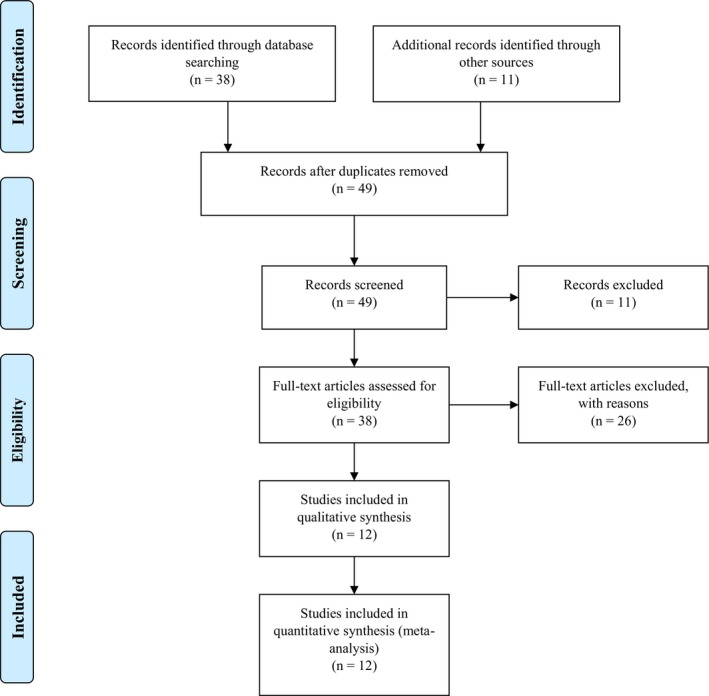
An overview of flow diagram showing study inclusion

A total of 49 studies were identified and screened for eligibility, with 38 records selected through database searching, and 11 articles retrieved from other sources. Overall, 12 studies met the inclusion criteria, as demonstrated in Figure 1. All included studies were RCTs published within the last five years, reporting on the impact of CoQ10 supplementation on CVD‐related outcomes in individuals with diabetes or metabolic syndrome. A total of eleven studies were excluded for lack of full‐text availability, whilst 25 records were omitted with reasons. For example, four studies^23^, ^24^, ^25^, ^26^ met the inclusion criteria but were excluded due to inconsistencies in data reporting. Other reasons for exclusion included reasons such as not having a clear study design, some studies were published outside the five‐year inclusion criteria, and others were excluded for not reporting on the impact of CoQ10 on individuals with diabetes or metabolic syndrome.

### 3.2 Study characteristics

All included articles were published in peer‐reviewed journals within the last five years, and in detail characteristic features of encompassed studies are displayed in Table 1. Briefly, this study comprised of a total of 650 participants with a mean age of 56.22 ± 10.93 years, with at least 50% of participants registered as males. Overall, 40% of studies reported findings from T2D patients, whilst 20% had diabetic retinopathy, 15% metabolic syndrome, 14% diabetic nephropathy, and 12% were prediabetic (Table 2). Treatment was distributed evenly, with 50% individuals receiving CoQ10 supplementation in included studies.

**Table 1:** Characteristic features of included studies and the reported impact of coenzyme Q_10_ (CoQ10) on cardiovascular disease‐related outcomes

Study	Country	Study size	Male, n (%)	Age (years)	CoQ dosage and duration	Main findings
Domanico et al, 2015^52^	Italy	68 patients with nonproliferative diabetic retinopathy (34 on CoQ10, 34 on placebo)	33 (48.5)	60.29 ± 8.51	CoQ10 (20 mg), with pycnogenol (50 mg) and vitamin E (30 mg) per day for 6 months	Treatment with antioxidants significantly reduced the levels of free radical species and central macular thickness in patients with diabetic retinopathy
Hosseinzadeh et al, 2015^54^	Iran	64 patients with type 2 diabetes (31 on CoQ10, 33 on placebo)	37 (57.8)	46.18 ± 7.96	200 mg per day for 12 weeks	Significant improvement in asymmetric dimethylarginine, serum nitrite and nitrate, as well as low‐density lipoprotein (LDL) and haemoglobin A1c (HbA1c) levels in CoQ10 compared to placebo group
Moazen et al, 2015^55^	Iran	52 patients with type 2 diabetes (26 per group)	28 (53.8)	51.73 ± 7.34	100 mg twice a day for 8 weeks	CoQ10 significantly reduced oxidative stress as measured by malondialdehyde (MDA) but did not impact fasting blood glucose (FPG), HbA1c and adiponectin levels
Pek et al, 2015^7^	Singapore	40 patients with diabetes (20 per group)	35 (87.5)	46.15 ± 12.01	150 mg per day for 12 weeks	Significantly reduced alanine aminotransferase and alkaline phosphatase, including regulated miRNAs (miR‐15a, miR‐21 and miR‐33a) that inhibit apoptotic and inflammatory pathways
Mehrdadi et al, 2016^27^	Iran	56 patients with type 2 diabetes (26 CoQ10, 30 placebo)	32 (57)	47.07 ± 7.55	200 mg per day for 12 weeks	Reduced HbA1c, body weight, body mass index (BMI), and adipolin levels. However, there was no significant alterations in FPG, fasting insulin and homeostasis model of assessment‐insulin resistance (HOMA‐IR) within or between CoQ10 and placebo groups
Raygan et al, 2016^30^	Iran	60 patients with metabolic syndrome (30 per group)	Not reported	62.9 ± 13.05	100 mg per day for 8 weeks	Although did not significantly impact FPG, lipid concentrations or inflammatory markers, CoQ10 had beneficial effects on serum insulin levels, HOMA‐IR, HOMA‐B and plasma total antioxidant capacity (TAC) concentrations
Gholnari et al, 2017^28^	Iran	50 patients with diabetic nephropathy (25 on CoQ10, 25 on placebo)	16 (32)	61.35 ± 10.56	100 mg per day for 12 weeks	Significantly reduced plasma MDA and advanced glycation end‐product levels compared with the placebo. However, CoQ10 had no significant impacts on FPG, lipid profiles, and matrix metalloproteinase‐2
Fallah et al, 2018^29^	Iran	60 patients with diabetic haemodialysis (30 per group)	40 (66.7)	62.10 ± 12.07	60 mg twice a day for 12 weeks	Induced beneficial effects on markers of insulin metabolism, but did not affect FPG, HbA1c, and lipid profiles
Heidari et al, 2018^11^	Iran	40 patients with diabetic nephropathy (20 on CoQ10, 20 on placebo)	Not reported	62.5 ± 31.82	100 mg per day for 12 weeks	Although did not significant affect gene expression of oxidized LDL, lipoprotein(a), glucose transporter (GLUT)‐1, transforming growth factor‐beta, CoQ10 markedly improved gene expression of peroxisome proliferator‐activated receptor‐gamma, interleukin‐1, and tumour necrosis factor‐alpha
Yen et al, 2018^56^	Taiwan	47 patients with type 2 diabetes (24 CoQ10, 23 placebo)	31(66)	60.57 ± 10.88	100 mg per day for 12 weeks	Increased antioxidant enzyme activity levels, reduced HbA1c levels and maintained HDL‐cholesterol levels
Yoo and Yum, 2018^57^	Korea	78 patients with prediabetes (39 per group)	57(73.08)	51.12 ± 7.75	200 mg per day for 8 weeks	CoQ10 significantly reduced HOMA‐IR no significant changes in FPG, insulin, and glycated haemoglobin
Kuhlman et al, 2018^8^	Denmark	35 overweight and obese individuals (18 on CoQ10, 17 on placebo)	22(62.9)	62.73 ± 1.71	2*200 mg per day for 8 weeks	Did not change muscle GLUT4 content, insulin sensitivity, or secretory capacity, but improved hepatic insulin sensitivity

**Table 2:** Summary of characteristic features of included studies

Characteristic features of included studies	Number	%
Total patients	650	100
Patients with diabetic retinopathy	128	20
Patients with diabetic nephropathy	90	14
Patients with metabolic syndrome	95	15
Patients with prediabetes	78	12
Patients with type 2 diabetes	259	40
Coenzyme Q_10_ supplementation	323	50
Placebo	327	50

### 3.3 Risk of bias assessment

**Figure 2:**
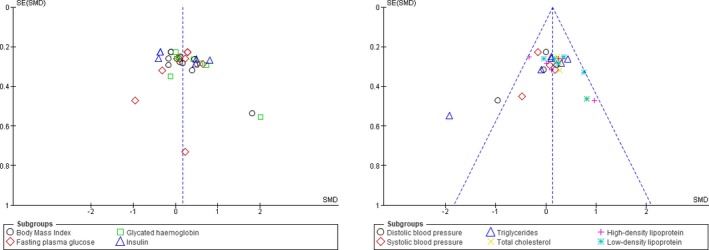
Funnel plot of glucose metabolic profiles and cardiovascular risk in diabetic patients on coenzyme Q10 supplements compared to those that received placebo showing no publication bias symmetry

The use of funnel plots showed perfect symmetry distribution which is indicative of no publication bias in the included studies (Figure 2). The risk of bias and quality of sixteen included studies was assessed by VM and KM, using a modified Downs and Black's checklist.^16^ The overall median score range of the 12 included studies was 19(14‐23) with seven of the rated good (19‐23 points) and the rest as fair (14‐18 points). Furthermore, all included studies had low reporting, internal and reporting bias with average median scores of 9(9‐10) out of a possible score of 11 (overall agreement 93%, kappa = 0, 87), 4(2‐6) out of a possible score of 7 (overall agreement 76%, kappa = 0, 50) and 5(3‐6) out of a possible score of 6 (overall agreement 93%, kappa = 0, 86), respectively. However, included studies scored poor on external validity with a median of 0(0‐3) out of a possible score of 3 (overall agreement 17%, kappa = −0,67).

### 3.4 Data synthesis

#### 3.4.1 The impact of CoQ10 supplementation on metabolic profiles

**Figure 3:**
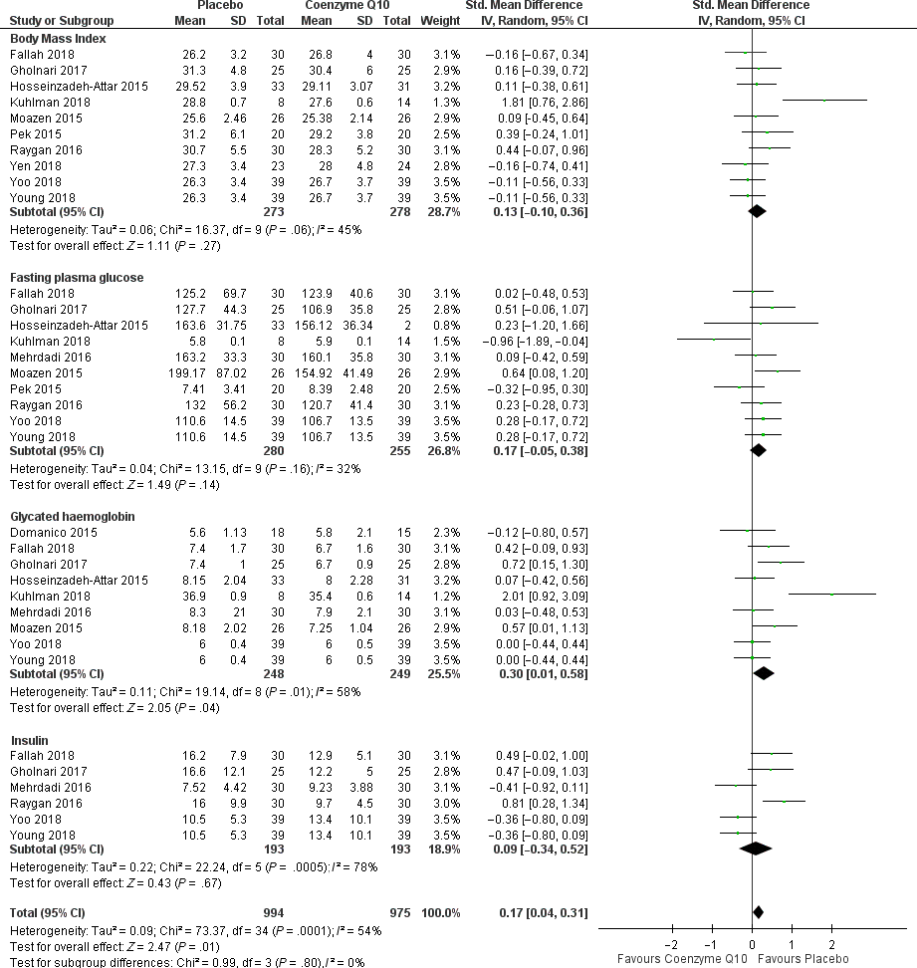
Statistical analysis data reporting on metabolic in diabetic patients on coenzyme Q10 supplements versus those on placebo

A total of 12 studies reported on glucose metabolism‐related profiles of included participants. Overall, the pooled estimates of metabolic profiles showed improved glucose metabolism in T2D patients on CoQ10 supplementation when compared to those on placebo ([SMD = 0.17, 95% CI (0.04; 0.31), Chi^2^ = 73.37 and *I* ^2^ = 54%, *P* = .0001). Despite high sources of heterogeneity, the test for subgroup differences indicated no significant effect (*P* = .80), thus no subgroup analysis was performed (Figure 3). Consistent with data of pooled estimates, qualitative data analysis showed that CoQ10 therapy could substantially improve other measured metabolic parameters when compared to those on placebo. For example, a study by Mehrdadi and colleagues demonstrated the positive effects of CoQ10 supplementation in reducing body weight and adipolin levels.^27^ Table 1 shows that the majority of studies demonstrated a positive outcome in controlling diabetes‐related features such as FPG and Hb1Ac.

#### 3.4.2 The impact of CoQ supplementation on cardiovascular risk parameters

**Figure 4:**
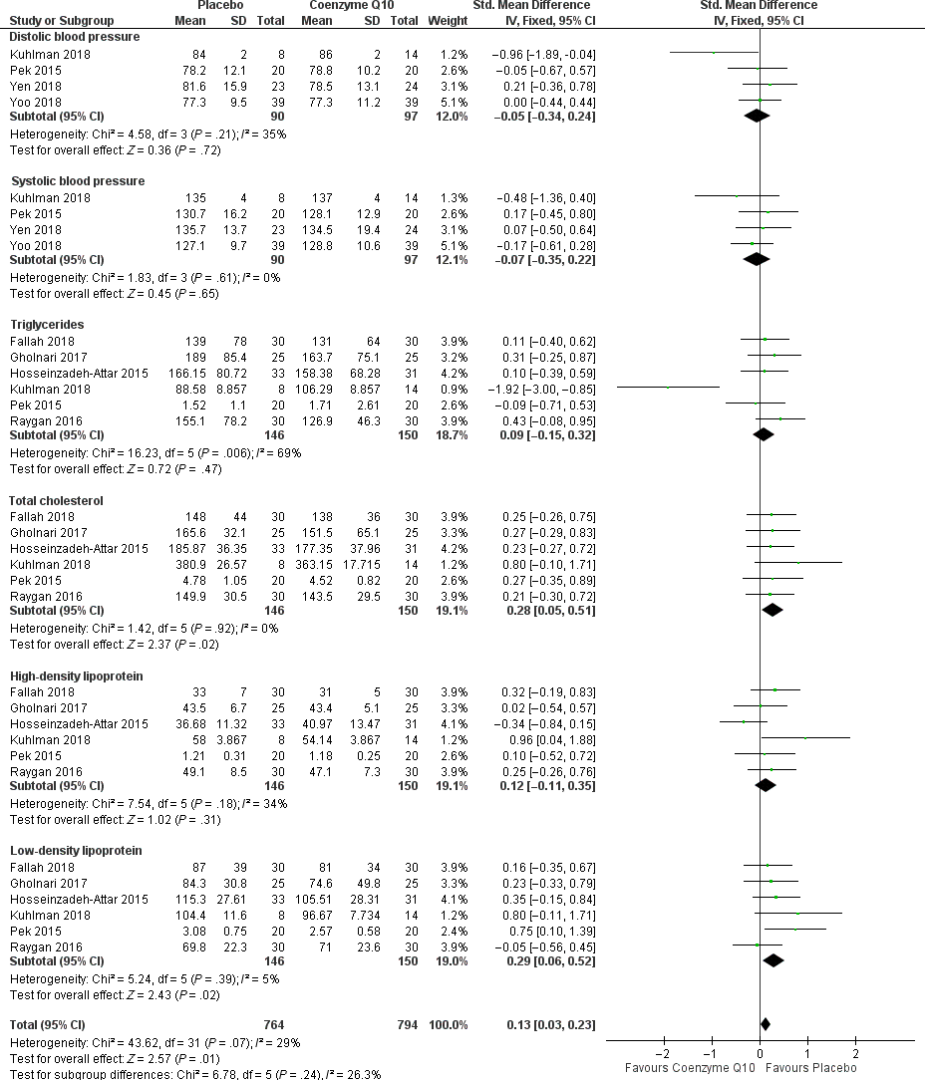
Statistical analysis data on cardiovascular disease risk profiles of diabetic patients on coenzyme Q10 supplements versus those that received placebo

It is now well established that dysregulated levels of lipid profiles in T2D or conditions of metabolic syndrome including LDL cholesterol, HDL, total cholesterol and triglycerides are associated with the development of CVDs. To establish a link between the lipid profiles and CVDs, a total of eight studies met the inclusion criteria. Interestingly, pooled estimates of cardiovascular risk effect measures showed a significant risk reduction in T2D patients on CoQ10 supplementation when compared to those on placebo [SMD = 0.13, 95% CI (0.03; 0.23], Chi^2^ = 43.62 and *I* ^2^ = 29%, *P* = .07. The test for subgroup differences suggested that there is an insignificant subgroup effect (*P* = .24), hence no subgroup analysis was performed based on the reported effect measures (Figure 4). It is of note that although the meta‐analysis supported the beneficial effects of CoQ10 on mediating lipid profiles, some of the included studies did not show any effect with CoQ10 supplementation on lipid profiles.^11^, ^28^, ^29^, ^30^ The quality of included evidence as well as the meta‐analysis summary of findings is reported in Table 3.

**Table 3:** An overview of the quality of included evidence as well as the meta‐analysis summary of findings

Coenzyme Q_10_ compared to placebo					
Patient or population: Adults patients with T2D					
Intervention: CoQ10					
Comparison: Placebo					
**Outcomes**	**Anticipated absolute effects** a ** (95% CI)**	**Relative effect 95% CI)**	**No of participants (studies)**	**Certainty of the evidence (GRADE)**	**Comments**
	**Risk with Placebo**	**Risk with CoQ10**			
Glucose metabolic profile measured using glycated haemoglobin (Hb1AC)	‐	The SMD in the intervention group was 0.30 lower (0.01 lower to 0.58 lower)	‐	419 (9 Randomised Control Trial studies)	⨁⨁⨁⨁ HIGH
Cardiovascular disease risk measured using total cholesterol	‐	The SMD in the intervention group was 0.28 lower (0.05 lower to 0.51 lower)	‐	236 (6 Randomised Control Trial studies)	⨁⨁⨁⨁ HIGH

Note

CI, Confidence interval; SMD, Standardised mean difference.

GRADE Working Group grades of evidence.

High certainty: We are very confident that the true effect lies close to that of the estimate of the effect.

Moderate certainty: We are moderately confident in the effect estimate: The true effect is likely to be close to the estimate of the effect, but there is a possibility that it is substantially different.

Low certainty: Our confidence in the effect estimate is limited: The true effect may be substantially different from the estimate of the effect.

Very low certainty: We have very little confidence in the effect estimate: The true effect is likely to be substantially different from the estimate of effect.

a The risk in the intervention group (and its 95% confidence interval) is based on the assumed risk in the comparison group and the relative effect of the intervention (and its 95% CI).

## 4 DISCUSSION

T2D and its interconnected metabolic complications such as obesity are of clinical significance because of their epidemic prevalence and contribution to the rapid rise of noncommunicable‐related deaths.^31^, ^32^ Dyslipidaemia remains the major characteristic feature of T2D, and its prevalence among diabetic patients may be high as 70% in some populations.^33^ Even worse, enhanced lipid deposits on the arterial wall in patients presenting with dyslipidaemia have been associated with the development and worsening of atherosclerosis.^34^ The latter is known to be the causal factor for virtually 80% of all deaths among diabetic patients.^35^ At present, complex mechanisms implicated in the development of atherosclerosis in a diabetic state have been described. For example, nonenzymatic glycosylation of lipids can interfere with normal physiological function by promoting the generation of oxidative stress, which is one of the major consequences associated with deteriorated cardiac function.

Oxidative stress, through enhanced production of reactive oxygen species, can affect endothelial nitric oxide (NO) availability, and thus in the process compromise vascular function.^36^ NO is broadly considered as one of the most vital molecules produced in the human body, and its acts as an essential regulator in a vast array of crucial physiological functions, especially the maintenance of vascular tone.^36^, ^37^ Research over the years has led to the identification of several ROS generating systems that could potentially be modulated in conditions of hyperglycaemia.^35^, ^38^, ^39^ However, therapeutic approaches currently being used to improve cardiovascular function, including maintenance of vascular tone such as aspirin and statins present with limited antioxidant properties to protect against oxidative stress.^40^, ^41^ Hence, exploration of antioxidant‐rich compounds such as CoQ10 remains a plausible strategy to improve cardiac function in a diabetic state. In agreement, a previous meta‐analysis of randomized controlled trials has supported the beneficial effects of CoQ10 supplementation on vascular endothelial function in humans.^10^ Others have reported on the meta‐analysis data showing contradictory results on the effect of CoQ10 on glycaemic control, lipid profile or blood pressure in patients with diabetes.^42^, ^43^, ^44^ Thus, highlighting the importance of updated evidence on this topic, especially understanding the impact of CoQ on metabolic and CVD‐related markers in the most vulnerable patients with T2D or metabolic syndrome.

The current systematic review and meta‐analysis summarized published evidence from twelve RCTs that explored the impact of CoQ10 therapy on metabolic and CVD‐related risk markers in patients with T2D or metabolic syndrome. Although did not statistically affect BMI or insulin levels, the pooled estimates of metabolic profiles showed that CoQ10 therapy could substantially improve other measured metabolic parameters when compared to those on placebo. The statistical significance results were especially seen with FPG and Hb1Ac. Overall, the current results suggest an enhanced potential for CoQ10 to improve basic metabolic parameters implicated in the deterioration of T2D. Moreover, the outcome showing that CoQ10 could not improve BMI or insulin levels is consistent with other reported findings elsewhere.^42^ Nevertheless, consistent with other pharmacological compounds like n‐acetyl cysteine and resveratrol, although could not significantly affect some metabolic parameters, their capacity to substantially enhance antioxidant capacity within the human system is important for attenuating oxidative stress and in the process show potential to protect the diabetic heart.^45^, ^46^, ^47^ Consistently, alpha‐lipoic acid, an organosulphur compound derived from caprylic acid that is increasingly explored for its beneficial effects as a dietary supplement with abundant properties, has been shown to provide limited amelioration against metabolic disturbances, but instead may protect against atherosclerosis and development of CVD when used in combination with exercise.^48^ Further, suggesting that perhaps the combination use of CoQ10 with exercise may provide even better protective effects against heart failure and associated complications, an aspect that is also increasingly explored elsewhere.^49^, ^50^

Nevertheless, presented data also showed that CoQ10 significantly reduced the overall CVD risk with a small effect size, with most studies reporting on the intervention between 8 and 12 weeks. In terms of effect measures for CVD, there was a significant reduction in only total cholesterol and LDL. It is important to note that although a previous study showed an effect in CVD risk measure such as systolic and diastolic blood pressure,^25^ the pooled estimates in the current review revealed that CoQ10 supplementation did not affect these parameters. However, the results on this CVD‐related outcome, including ejection fraction, are inconclusive due to the limitation of RCTs that have explored these important CVD‐related outcomes. Interestingly, although a formal analysis was not done on the effect of CoQ10 on oxidative stress parameters due to heterogeneous nature of reported data, it was evident that CoQ10 supplementation consistently improved the antioxidant capacity in patients, thus resulting in reduced oxidative stress markers like plasma malondialdehyde and advanced glycation end‐product levels. Of particular interest was also the reported ability of CoQ10 to enhance NO bioavailability by one of the included studies, consistent with beneficial effects in improving the antioxidant status of T2D patients.^30^ Further suggesting that CoQ10 exerts an enhanced effect in improving vascular tone as well as endothelial function, as reported previously.^10^ This is especially important since it is acknowledged that endothelial dysfunction is implicated in the deterioration of cardiac function, especially in conditions of metabolic syndrome.^51^ However, more studies are required to assess the impact of CoQ10 supplementation of oxidative stress parameters, in correlation to CVD risk outcomes in diabetic patients. Similarly, long‐term intervention with CoQ10 could be of interest to explore, since except for one study,^52^ the majority of RCTs were done for a period of three months or less.

## 5 CONCLUDING STATEMENT

CoQ10 is a component of the electron transport chain that is vital in aerobic cellular respiration. In addition, this lipophilic compound can act as a strong antioxidant by blocking protein, lipid and DNA oxidation. In turn, experimental data and clinical trials have increased reporting on the impact of CoQ10 on CVD risk in patients with metabolic diseases. On the other hand, it is well understood that dietary supplementation with CoQ10 leads to enhanced ubiquinol‐10 levels within circulating lipoproteins, and this consequence is essential for blocking oxidative stress, especially the initiation of lipid peroxidation.^53^ In support of this fact, the current systematic review and meta‐analysis demonstrated that in addition to improving important metabolic profiles, CoQ10 supplementation could lower CVD risk by reducing total cholesterol and LDL levels in patients presenting with diabetes or metabolic syndrome. Thus, more research is needed exploring the clinical use of CoQ10 to maintain metabolic diseases, which is important to prolong the lives of diabetic patients. This is consistent with the identified limitations, like assessing its synergistic effect when combined with current anti‐diabetic agents, as well as testing its overall impact on broader spectrum of CVD‐risk‐related outcomes such as ejection fraction.

## CONFLICT OF INTEREST

The authors declare no conflict of interest.

## ETHICS APPROVAL

This review manuscript summarizes and informs of already published studies and thus does not require ethical approval.

## AUTHORS' CONTRIBUTIONS

PVD and LT conceived the study and drafted the original manuscript and original draft; VM and KM extracted the data and involved in study appraisal; TMN and BBN statistically analysed the data; PVD, TMN, PO, SS, VM, KM, BBN, JL, CJF and LT involved in the writing and final approval of the manuscript.

## Data Availability

All data used to support the findings of this study are included within the article. Raw data can be available on request after publication.
